# Supplementary material for: Microtubule flexibility, microtubule-based nucleation and ROP pattern co-alignment enhance protoxylem microtubule patterning
Source: Quant Plant Biol. 2025 Jan 27;6:e2. doi: 10.1017/qpb.2024.17 (PMC11811878; doi:10.1017/qpb.2024.17)
Supplement: Jacobs et al. supplementary material [file S2632882824000171sup001.pdf]

Supplementary information for: Microtubule flexibility, microtubule-based nucleation and ROP pattern co-alignment enhance protoxylem microtubule patterning

Bas Jacobs, Marco Saltini, Laura Filion, Jaap Molenaar, Eva E. Deinum

Contents

1 Supplementary figures **2**

Appendices **14**

A Simulation details and parameter values **14**
A.1 Target nucleation rate 14

B Semiflexible microtubules **16**

C Persistence length calculations **17**

D Summary statistics **18**

References **19**

1 Supplementary figures

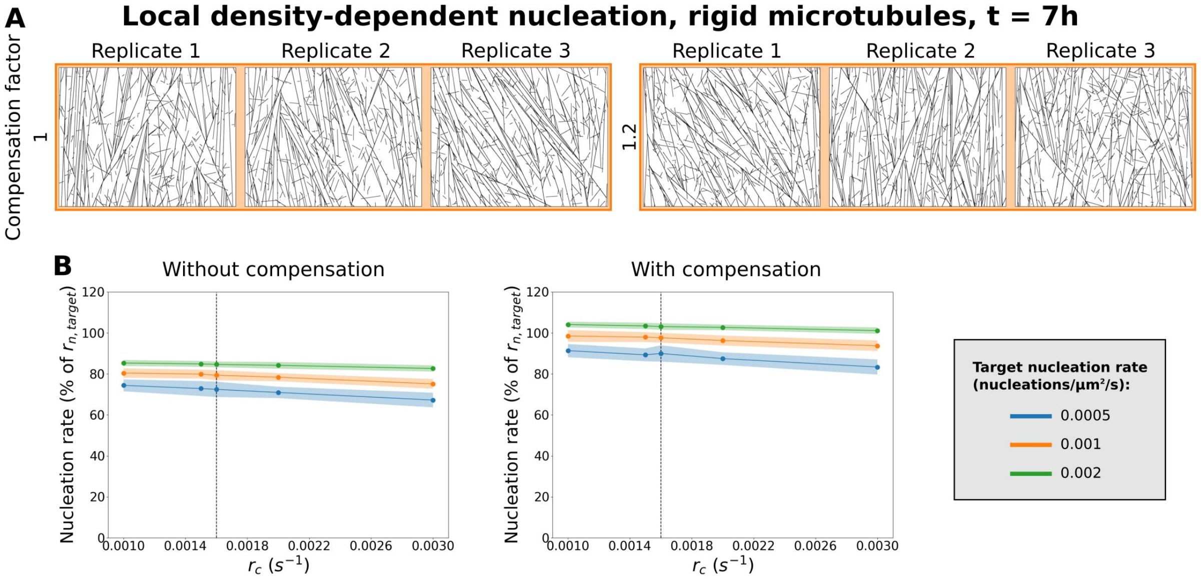

Figure S.1: A twenty percent compensation on the nucleation complex appearance rate keeps the achieved nucleation rate close to the target. (A) Snapshots at $t = 7h$ from array simulations without bands with locally saturating nucleation with and without compensation. (B) Realised nucleation rate, calculated over a 200 s measurement interval, as a percentage of the target rate ($r_{n,target}$). Quantities in (B) were calculated from 100 simulations. Lines indicate the average and shaded areas the standard deviation.

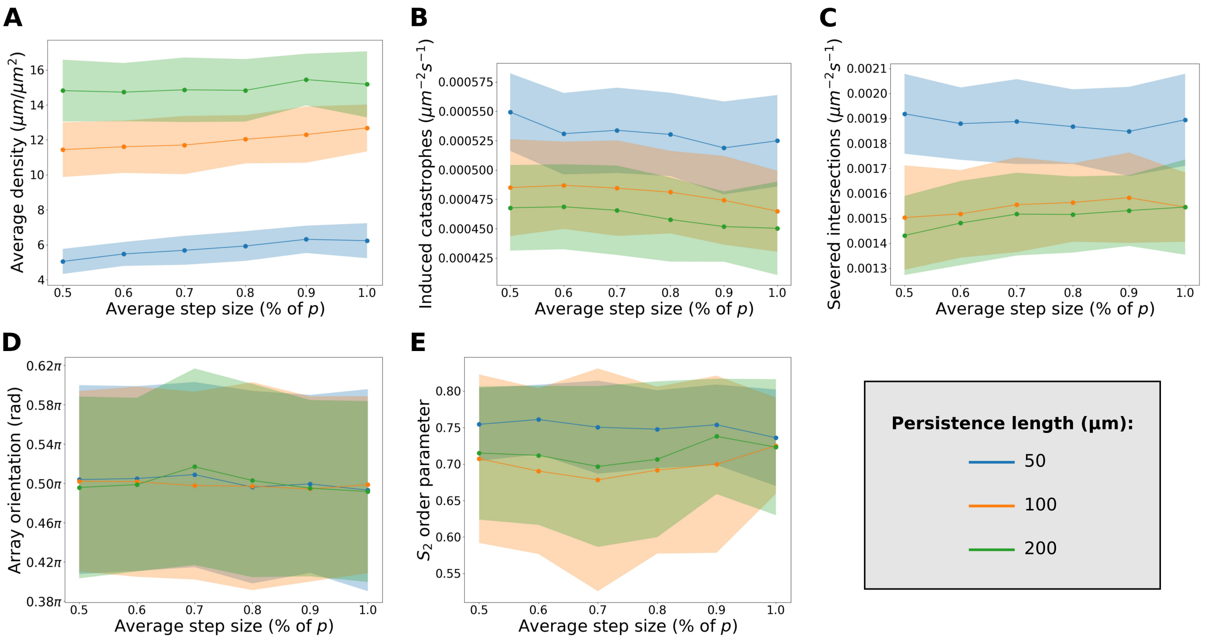

Figure S.2: Influence on array behaviour of different step sizes for the same persistence length. (A–E) Quantification of array state and simulation processes at $t = 7h$ from simulations without bands using isotropic nucleation for various difference persistence lengths and deflection step sizes. Simulations used the default cylindrical geometry and were initiated with seeded nucleations as described in Appendix A. (A) Average microtubule density. (B) Overall number of induced catastrophes per unit area per second. (C) Overall number of intersection severing events per unit area per second. (D) Average array orientation. (E) S_2 order parameter, showing degree of alignment. (A,D,E) Quantities measured at $t = 7h$ intervals. (B,C) Quantities averaged over the last 200 s measurement interval. Quantities in (A–E) were calculated from 100 simulations. Lines indicate the average and shaded areas the standard deviation. Step sizes ($\bar{l}$) are expressed as a percentage of the persistence length l_p.

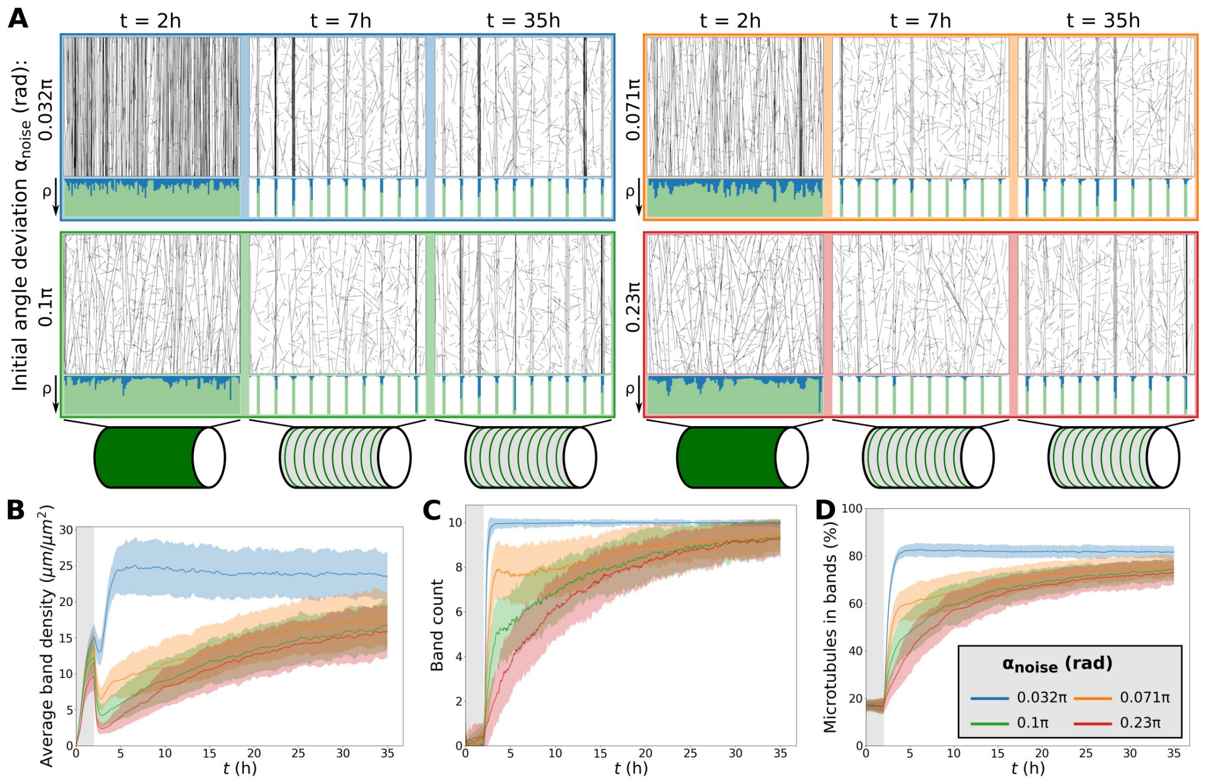

Figure S.3: Isotropic nucleation allows fast band formation for sufficiently co-aligned arrays. (A) Snapshots from protoxylem simulations with isotropic nucleation using starting arrays obtained with different values for α_{noise}. Histograms below snapshots showing local microtubule density ρ share the same axis within a time series. For the different time series, the ρ-axis ranges from 0 to 75, 56, 78, and 60 $\mu m/\mu m^2$, respectively. (B) Average microtubule density in the band regions. (C) Number of populated bands, defined as bands with a microtubule density greater than three times the average density in the gaps. (D) Percentage of the total microtubule length residing in the bands. Quantities in (B–D) were calculated from 100 simulations. The band formation phase starts at $t = 2h$, i.e., at the end of the grey area. Lines indicate the average and shaded areas the standard deviation.

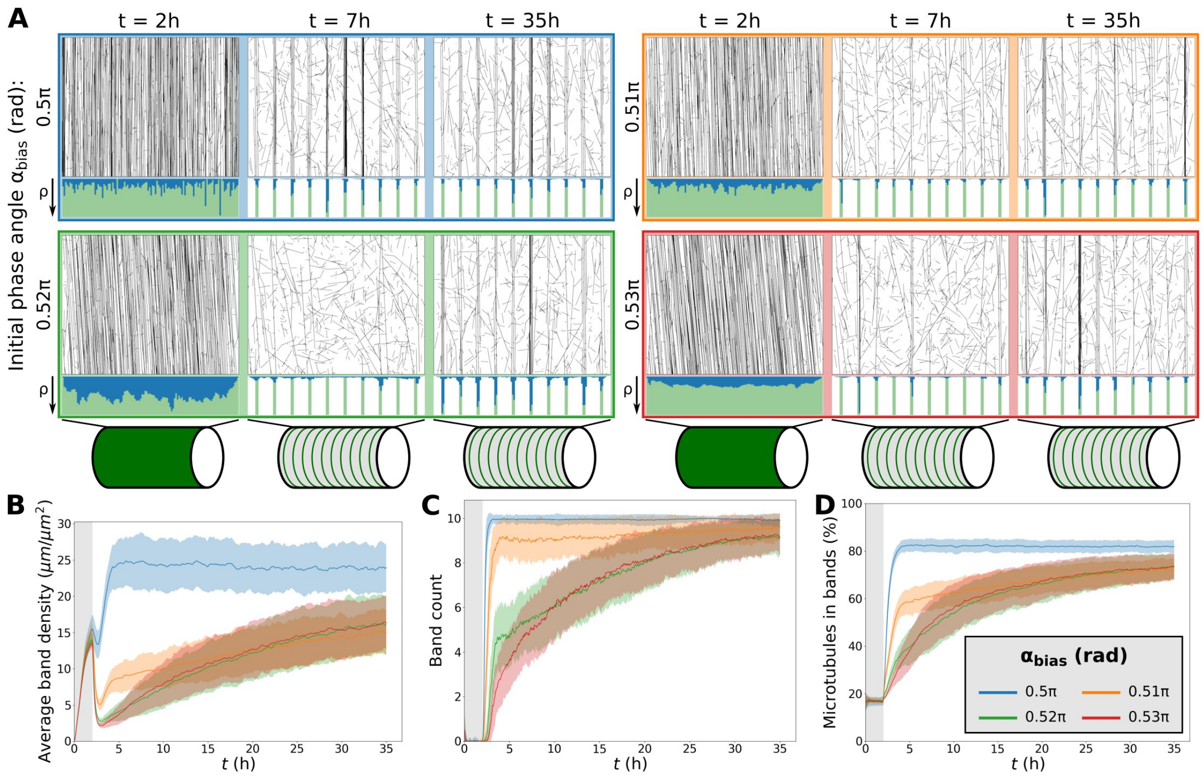

Figure S.4: Fast protoxylem patterning is sensitively dependent on co-alignment between microtubules and the underlying pattern. (A) Snapshots from protoxylem simulations with isotropic nucleation using starting arrays with different bias angles α_{bias} in the first half hour with only minor deviations ($\alpha_{noise} = 0.032\pi$ rad). Histograms below showing local microtubule density ρ share the same axis within a time series. For the different time series, the ρ-axis ranges from 0 to 77, 68, 26, and 64 $\mu m/\mu m^2$, respectively. (B) Average microtubule density in the band regions. (C) Number of populated bands, defined as bands with a microtubule density greater than three times the average density in the gaps. (D) Percentage of the total microtubule length residing in the bands. Quantities in (B–D) were calculated from 100 simulations. The band formation phase starts at $t = 2h$, i.e., at the end of the grey area. Lines indicate the average and shaded areas the standard deviation.

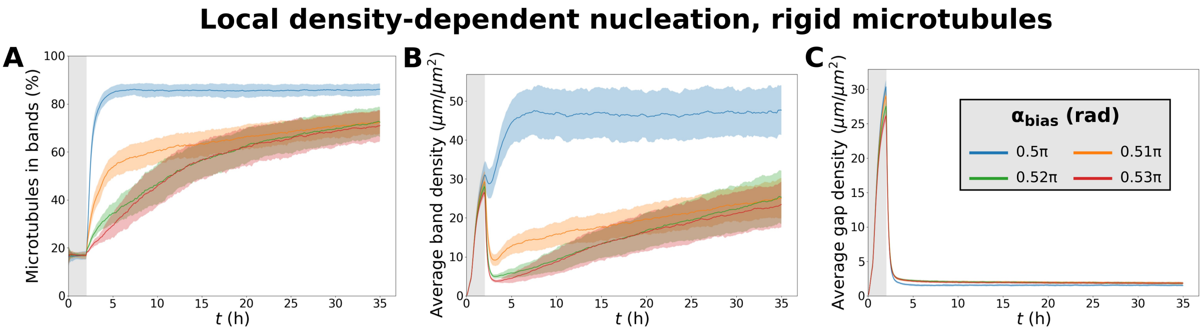

Figure S.5: Band formation is due to density loss in gaps regions. Charts correspond to simulations from Fig. 2 from the main text. (A) Percentage of the total microtubule length residing in the bands. (B) Average microtubule density in the band regions. (C) Average microtubule density in the gap regions.

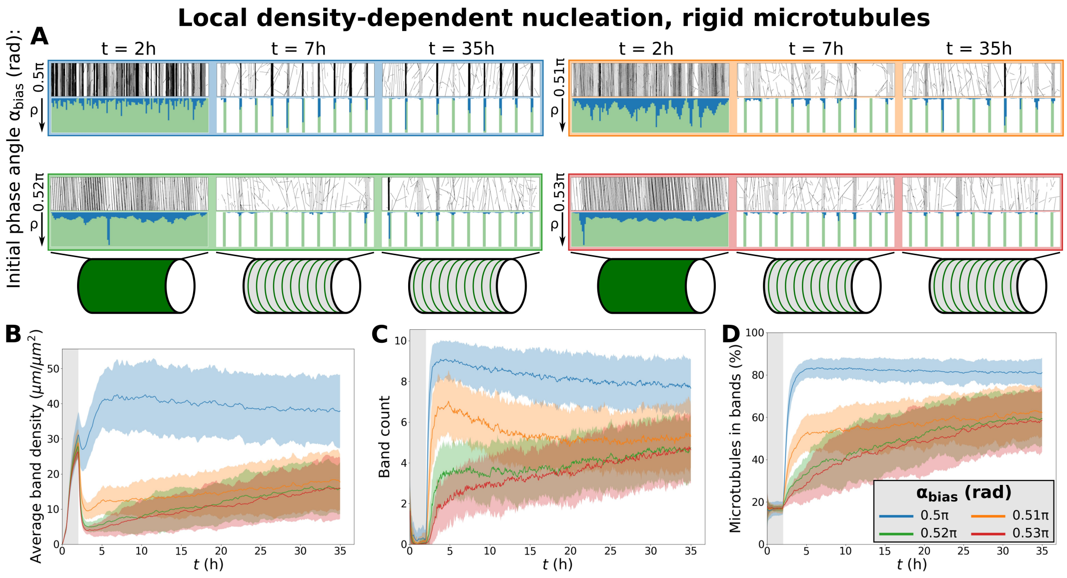

Figure S.6: Protoxylem simulations with rigid microtubules for a $2\mu m$ cell radius. (A) Snapshots from protoxylem simulations using starting arrays with bias angles α_{bias} of 0.5π (90°), 0.51π (91.8°), 0.52π (93.6°), and 0.53π (95.4°) in the first half hour with only minor deviations ($\alpha_{noise} = 0.032\pi$ rad). Histograms below showing local microtubule density ρ share the same axis within a time series. For the different time series, the ρ-axis ranges from 0 to 196, 93, 149, and 150 $\mu m/\mu m^2$, respectively. (B) Average microtubule density in the band regions. (C) Number of populated bands, defined as bands with a microtubule density greater than three times the average density in the gaps. (D) Percentage of the total microtubule length residing in the bands. Quantities in (B–D) were calculated from 100 simulations. The band formation phase starts at $t = 2h$, i.e., at the end of the grey area. Lines indicate the average and shaded areas the standard deviation.

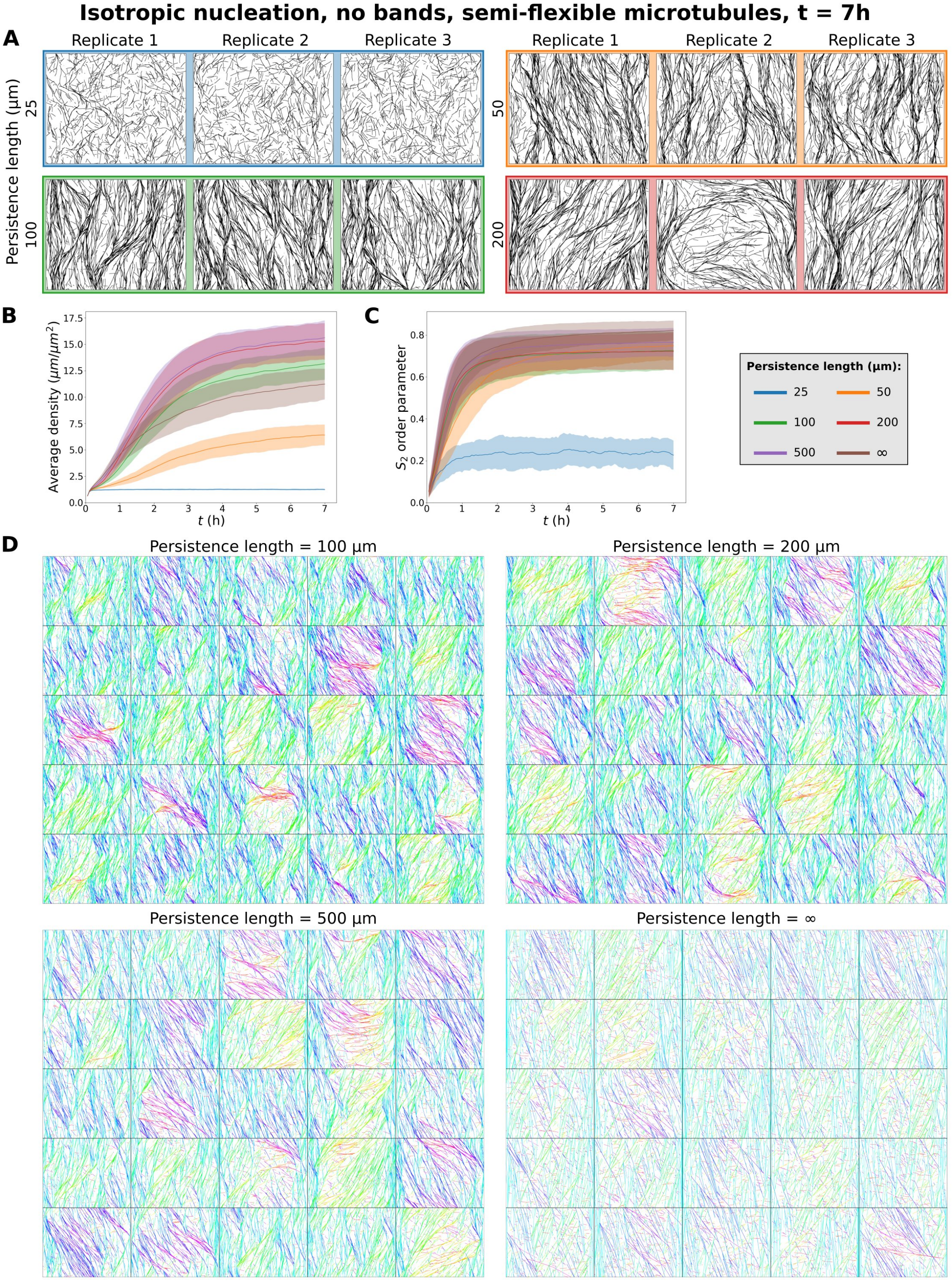

Figure S.7: Semiflexible microtubules result in low densities and failure to align at low persistence lengths and reduced global alignment at intermediate persistence length. (A) Snapshots at $t = 7h$ from array simulations without bands with isotropic nucleation for different microtubule persistence lengths. (B) Average microtubule density. (C) S_2 order parameter, showing degree of alignment. Quantities in (B–C) were calculated from 100 simulations. Lines indicate the average and shaded areas the standard deviation. (D) Snapshots at $t = 7h$ from 25 independent array simulations with isotropic nucleation without bands for different persistence lengths, and rigid microtubules (infinite persistence length). Microtubule segments are coloured by their orientation. N.B., the faded colouring for larger persistence lengths is largely due to bundles drawn as a single line.

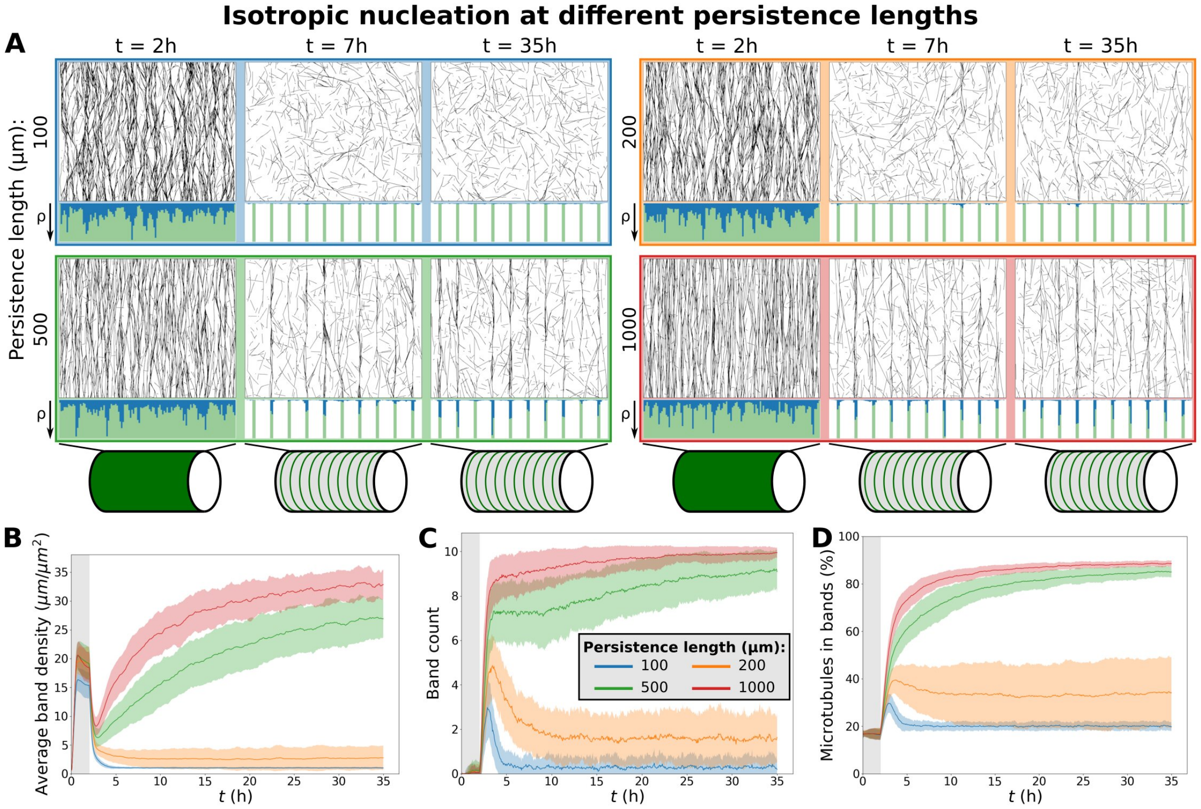

Figure S.8: Band maintenance under isotropic nucleation requires high persistence length. (A) Snapshots from protoxylem simulations with isotropic nucleation for different microtubule persistence lengths. Histograms below showing local microtubule density ρ are plotted on the same scale within a time series. For the different time series, the ρ-axis ranges from 0 to 50, 53, 63, and 69 $\mu m/\mu m^2$, respectively. Starting arrays were obtained with transverse nucleations in the first half hour ($\alpha_{bias} = 0.5\pi$, $\alpha_{noise} = 0.032\pi$ rad). (B) Average microtubule density in the band regions. (C) Number of populated bands, defined as bands with a microtubule density greater than three times the average density in the gaps. (D) Percentage of the total microtubule length residing in the bands. Quantities in (B–D) were calculated from 100 simulations. The band formation phase starts at $t = 2h$, i.e., at the end of the grey area. Lines indicate the average and shaded areas the standard deviation.

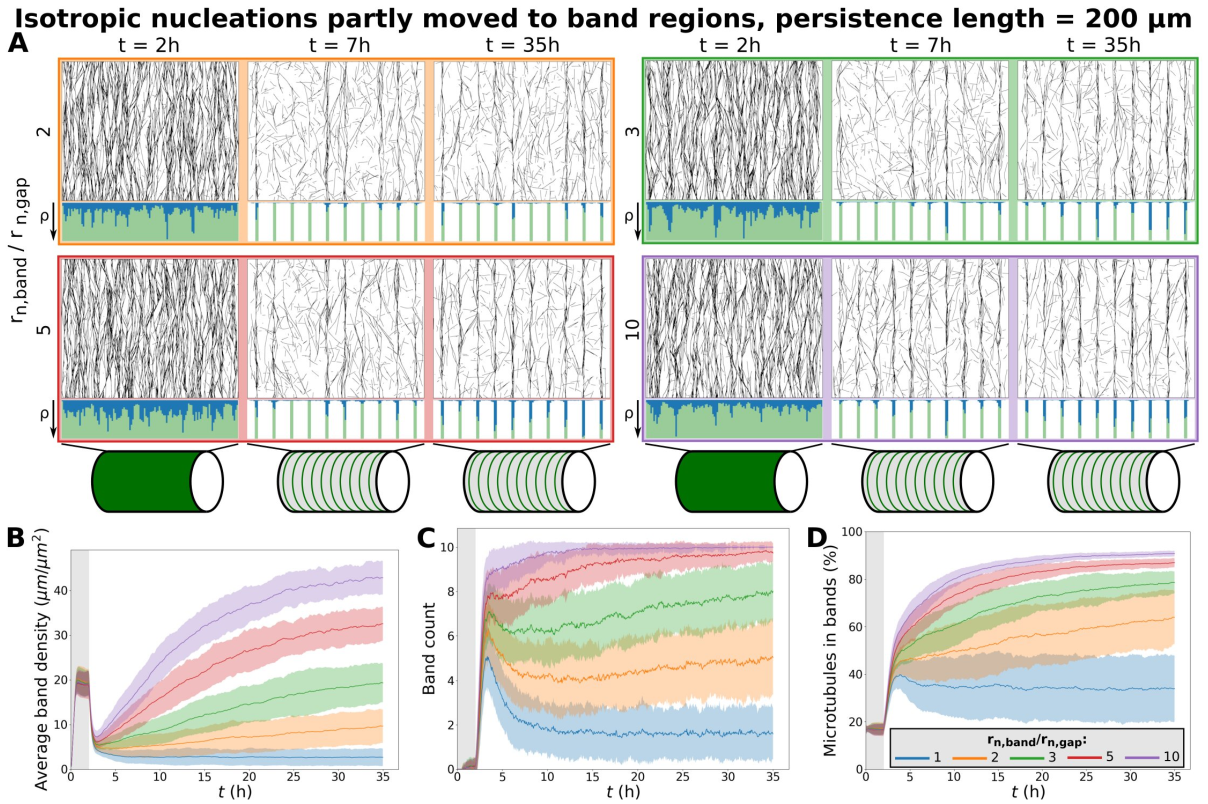

Figure S.9: With isotropic nucleation, a shift of nucleations from gaps to bands can maintain band density. (A) Snapshots from protoxylem simulations with a fraction of isotropic nucleations moved from gaps to bands, keeping the overall nucleation rate the same. The persistence length used was 200 μm. Starting arrays were obtained with transverse nucleations in the first half hour ($\alpha_{bias} = 0.5\pi$, $\alpha_{noise} = 0.032\pi$ rad). Histograms below showing local microtubule density ρ are plotted on the same scale within a time series. For the different time series, the ρ-axis ranges from 0 to 80, 62, 57, and 78 $\mu m/\mu m^2$, respectively. (B) Average microtubule density in the band regions. (C) Number of populated bands, defined as bands with a microtubule density greater than three times the average density in the gaps. (D) Percentage of the total microtubule length residing in the bands. Quantities in (B–D) were calculated from 100 simulations. The band formation phase starts at $t = 2h$, i.e., at the end of the grey area. Lines indicate the average and shaded areas the standard deviation.

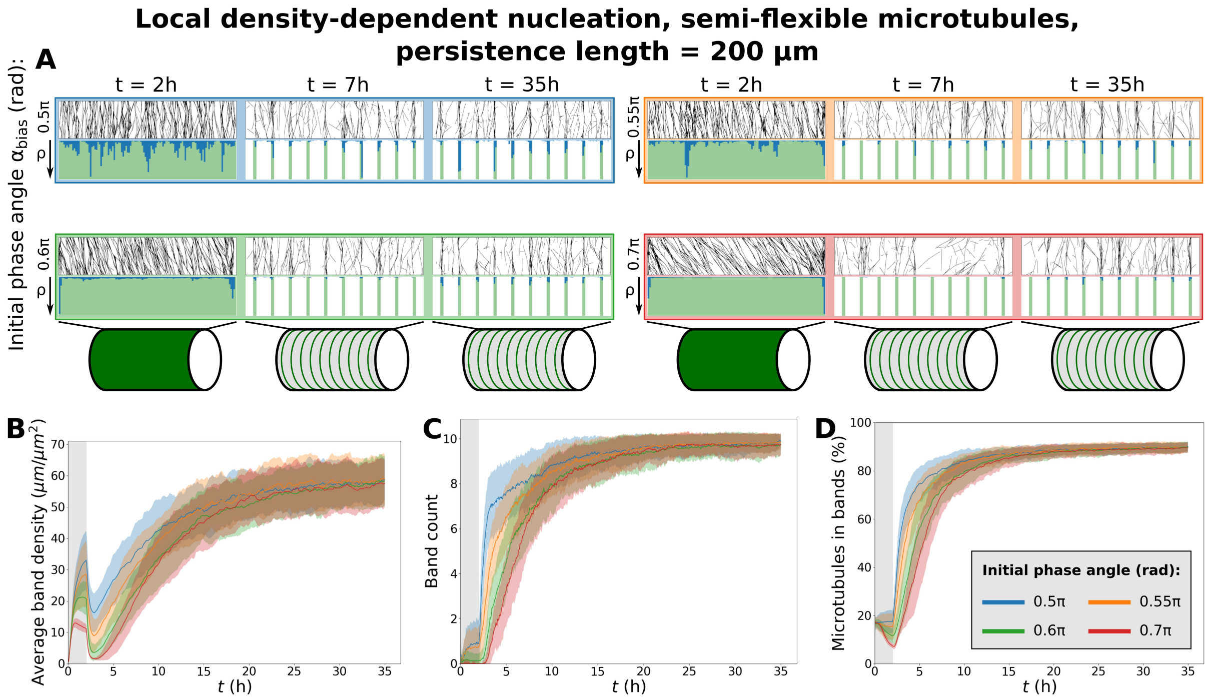

Figure S.10: Protoxylem simulations with semiflexible microtubules for a $2\mu m$ cell radius. (A) Snapshots from protoxylem simulations for $p = 200\ \mu m$ with locally saturating nucleation using starting arrays with different bias angles α_{bias} in the first half hour with only minor deviations ($\alpha_{noise} = 0.032\pi$ rad). Histograms below showing local microtubule density ρ are plotted on the same scale within a time series. For the different time series, the ρ-axis ranges from 0 to 171, 364, 541, and 926 $\mu m/\mu m^2$, respectively. (B) Average microtubule density in the band regions. (C) Number of populated bands, defined as bands with a microtubule density greater than three times the average density in the gaps. (D) Percentage of the total microtubule length residing in the bands.

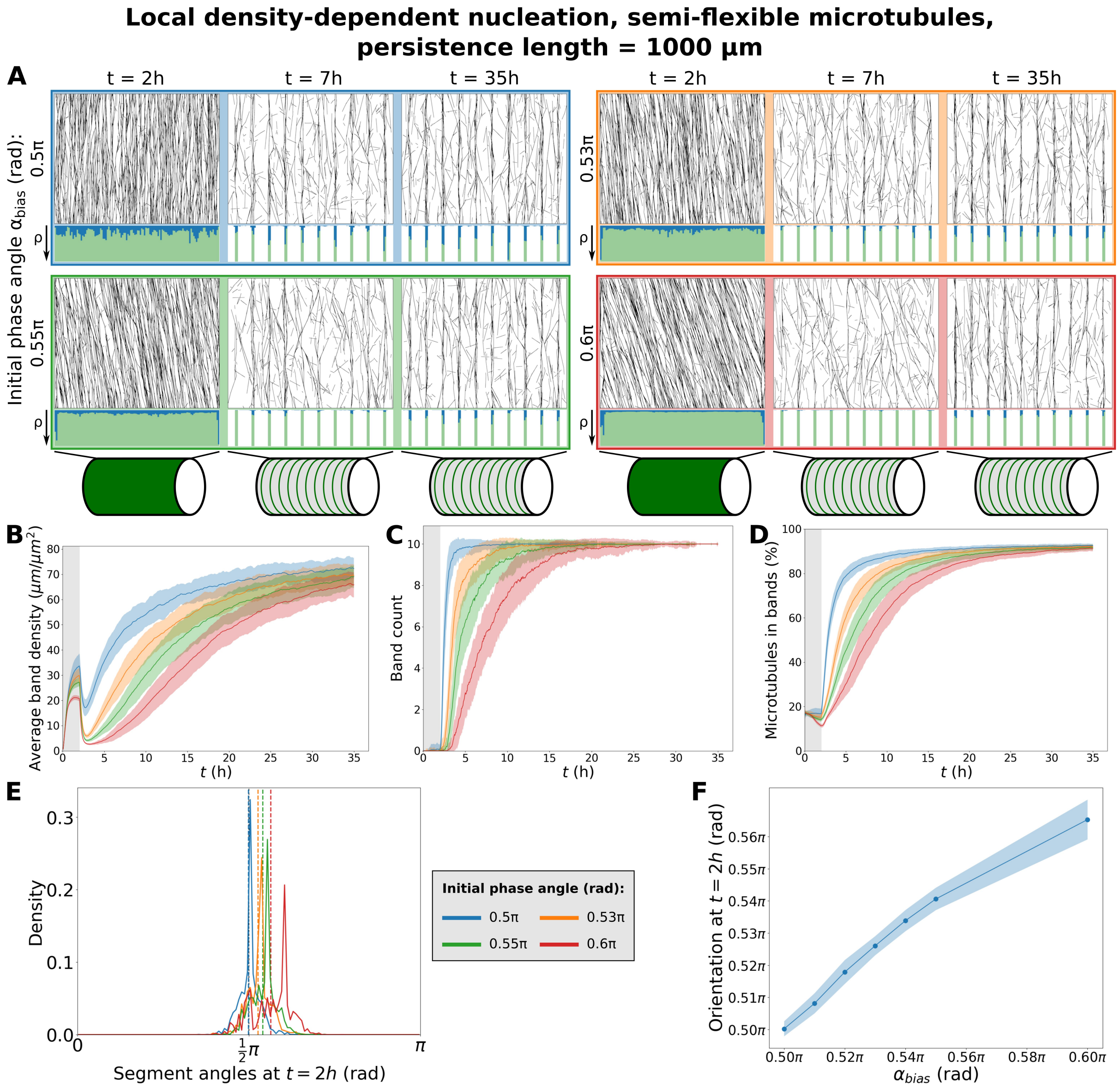

Figure S.11: Simulations with locally saturating nucleation and $l_p = 1000\ \mu m$ (Fig. 1 in the main text), using starting arrays with different bias angles α_{bias} in the first half hour with only minor deviations ($\alpha_{noise} = 0.032\pi$ rad). (A) Simulation snapshots. Histograms below showing local microtubule density ρ are plotted on the same scale within a time series. For the different time series, the ρ-axis ranges from 0 to 160, 246, 284, and 428 $\mu m/\mu m^2$, respectively. (B) Average microtubule density in the band regions. (C) Number of populated bands, defined as bands with a microtubule density greater than three times the average density in the gaps. (D) Percentage of the total microtubule length residing in the bands. (E) Distribution of microtubule segment angles, weighted by segment length, at $t = 2h$ from the individual example simulations shown in (A). Dashed lines indicate the the overall array orientation. (F) Average array orientation at $t = 2h$ as a function of the bias angle in the initiation phase. Quantities in (B–D), and (J) were calculated from 100 simulations. The band formation phase starts at $t = 2h$, i.e., at the end of the grey area. Lines indicate the average and shaded areas the standard deviation.

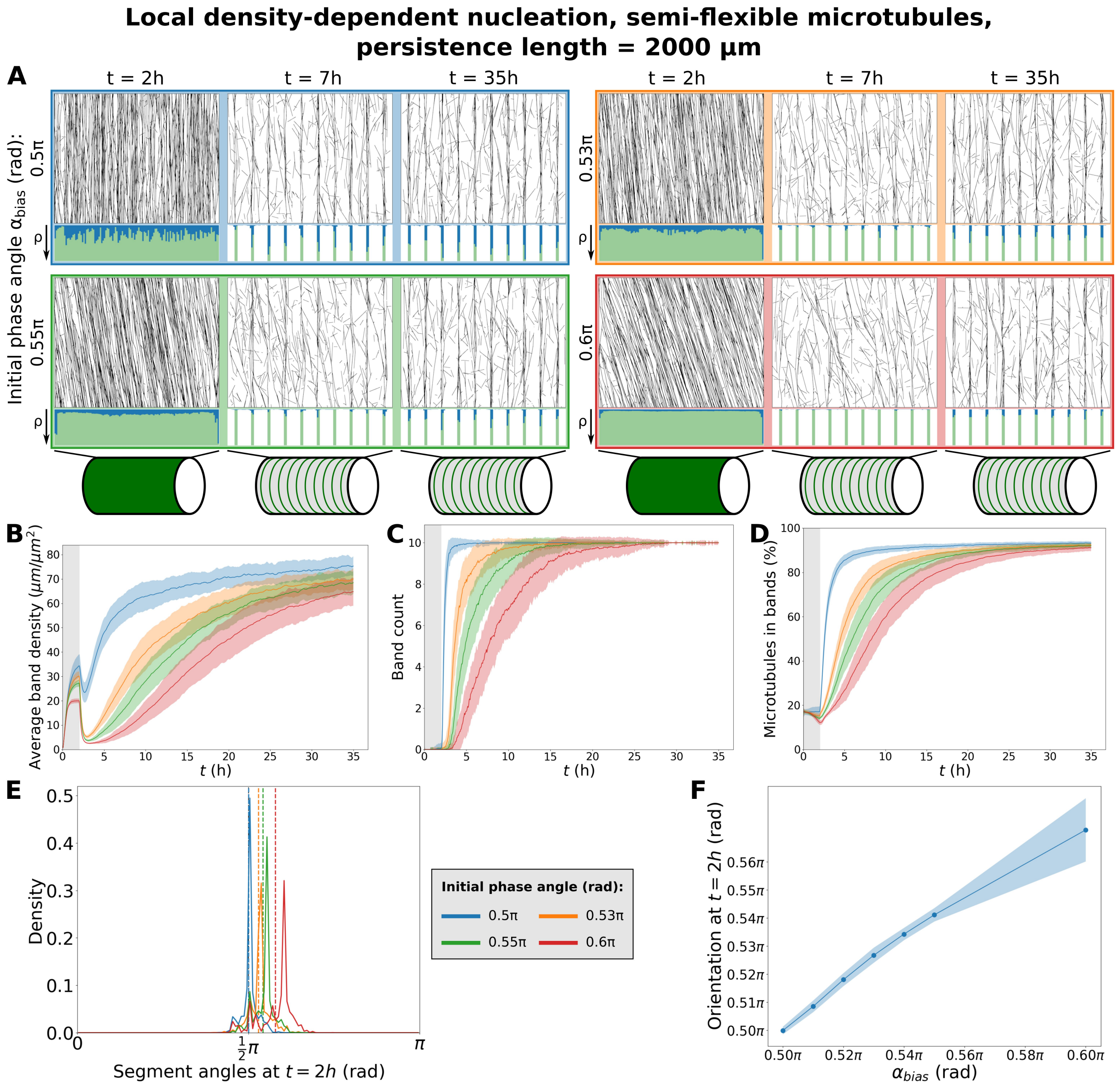

Figure S.12: Simulations with locally saturating nucleation and $l_p = 2000\ \mu m$ (Fig. 1 in the main text), using starting arrays with different bias angles α_{bias} in the first half hour with only minor deviations ($\alpha_{noise} = 0.032\pi$ rad). (A) Simulation snapshots. Histograms below showing local microtubule density ρ are plotted on the same scale within a time series. For the different time series, the ρ-axis ranges from 0 to 126, 218, 233, and 395 $\mu m/\mu m^2$, respectively. (B) Average microtubule density in the band regions. (C) Number of populated bands, defined as bands with a microtubule density greater than three times the average density in the gaps. (D) Percentage of the total microtubule length residing in the bands. (E) Distribution of microtubule segment angles, weighted by segment length, at $t = 2h$ from the individual example simulations shown in (A). Dashed lines indicate the the overall array orientation. (F) Average array orientation at $t = 2h$ as a function of the bias angle in the initiation phase. Quantities in (B–D), and (J) were calculated from 100 simulations. The band formation phase starts at $t = 2h$, i.e., at the end of the grey area. Lines indicate the average and shaded areas the standard deviation.

Supplementary Texts

A Simulation details and parameter values

Simulations were performed with an extended version of 'CorticalSim' [12], a well established and fast two-dimensional microtubule simulation platform [1–3, 11, 13, 14]. To account for the possibility that some katanin severing events at crossover intersections may have been interpreted as induced catastrophes [3] in the original experiments and analysis by Dixit and Cyr [4], the probability P_{cat} that collisions at large angles result in catastrophes was lowered compared to previous studies [2, 14]. Contrary to those studies, we included katanin severing by default, with a rate of $r_x = 0.023\ s^{-1}$ per crossover, similar to values found in experiments [7, 10, 15].

Protoxylem simulations were performed with $1\ \mu m$ wide band regions separated by $5\ \mu m$ wide gap regions as in Schneider et al. [11]. Simulations started with a $2h$ initiation phase without bands followed by a $5h$ or $33h$ band formation phase in which the catastrophe rate in the gap regions was increased by a factor $f_{cat} = 3$, which in experimental observations tends to be achieved or exceeded during a substantial part of the patterning process [11]. In the first $0.5h$ of the initiation phase, nucleations were distributed uniformly and given an angle α_{bias}, with a small amount of normally distributed noise, with a standard deviation α_{noise} of 0.032π rad. For simulations with rigid microtubules, this biased phase had almost no collisions that could lead to induced catastrophes or crossovers. To compensate, the nucleation rate for this phase was reduced by a factor 4 for simulations with rigid microtubules. The remainder of the initiation phase used the same nucleation mode as the band formation phase. Simulations without bands were run for $7h$ starting from an empty array with additional isotropic nucleations added at the beginning speed up the population of the array. These *'seeded' nucleations* were added at a density of $0.1\ \mu m^{-2}s^{-1}$ and a rate of $0.003\ s^{-1}$ as in Lindeboom et al. [6]. See Table 1 for default parameter values.

A.1 Target nucleation rate

For calculating nucleation parameters, we aimed at an overall target nucleation rate $r_{n,target}$ for homogeneous arrays of 0.001 nucleations $\mu m^{-2}s^{-1}$, consistent with previous work [2, 5, 11]. As a first estimate, we assumed that the fraction of unbound nucleations in a fully populated array would be negligible.

From there, we calculated back to a parameter $r_{n,max}$ (the nucleation rate when all nucleation complexes are free and all nucleations microtubule-bound). For this, we used the factor difference f_{rn} between the nucleation rate when all complexes are available and the target nucleation rate. We estimated this rate from the ratio between microtubule-associated appearances in nearly empty oryzalin-treated arrays (0.013 appearances $\mu m^{-2}s^{-1}$) and total, mostly microtubule-associated, appearances in established arrays (0.0037 appearances $\mu m^{-2}s^{-1}$) that were measured in [5]. This approach gives:

$$r_{n,max} = f_{rn} \cdot r_{n,target} = \frac{0.013}{0.0037} \cdot 0.001 = 0.0035\mu m^{-2}s^{-1}. \quad (1)$$

With a rejection probability of 0.76 for microtubule-bound nucleations, we computed a required maximum appearance rate of:

$$r_{app} = 0.0035/(1 - 0.76) = 0.015\mu m^{-2}s^{-1} \quad (2)$$

Table 1: Default parameter values used in the simulations.

Parameter	Value	Unit	Description	Source
Simulation domain				
H	60	μm	Domain length	[11]
W	$7.5 \cdot 2\pi$	μm	Domain circumference	[11]
Dynamic instability				
v^+	0.05	$\mu m/s$	Growth speed	[11]
v^-	0.08	$\mu m/s$	Shrinkage speed	[11]
v^{tm}	0.01	$\mu m/s$	Minus end retraction speed	[11]
r_c	0.0016	s^{-1}	Catastrophe rate (bands)	[11]
r_r	0.001	s^{-1}	Rescue rate	[11]
Microtubule interaction				
θ_c	40°		Collision angle where outcome switches from bundling to crossover or induced catastrophe	[4]
P_{cat}	0.09		Induced catastrophe probability for large-angle encounters	[3]
r_x	0.023	s^{-1}	Crossover severing rate per crossover	
Nucleation				
r_n	0.001	$\mu m^{-2}s^{-1}$	Nucleation rate for isotropic nucleation	[11]
$r_{n,target}$	0.001	$\mu m^{-2}s^{-1}$	Nucleation rate for a populated homogeneous array with locally saturating nucleation	[11]
r_{app}	0.018	$\mu m^{-2}s^{-1}$	Nucleation complex appearance rate when all nucleation complexes are free.	
$t_{occupied}$	60	s	Time a nucleation complex stays occupied after a nucleation	[9]
Band formation				
f_{cat}	3		Factor increased catastrophe rate in gaps	
Band width	1	μm	Width of band regions	[11]
Gap width	5	μm	Width of gap regions	[11]
Semiflexible microtubules				
l_p	200	μm	Persistence length	
$\bar{l}$	$0.01 l_p$	μm	Average deflection step size	
q	0.1°		Minimum deflection angle	
θ_b	10°		Maximum bundle tracking angle	

We further used a fixed duration $t_{occupied}$ of 60s during which a nucleation complex remains occupied upon nucleation, based on an average of 58.9s observed in experimental work [9]. Using our estimates for parameters $r_{n,target}$, $r_{n,max}$, and $t_{occupied}$, we calculated the remaining parameter N_{tot}. At nucleation rate $r_{n,target}$, a number of $N_{occ,target}$ nucleation complexes are

expected to be occupied, following:

$$r_{n,target} = r_{n,max} \frac{N_{tot} - N_{occ,target}}{N_{tot}}, \tag{3}$$

where N_{tot} is the total number of nucleation complexes. Using this expression and the fact that the number of occupied complexes depends on the duration of occupancy $t_{occupied}$ and the rate at which they become occupied (i.e., the global nucleation rate), we found the following expression for N_{tot}:

$$N_{tot} = \frac{r_{n,max} \cdot N_{occ,target}}{r_{n,max} - r_{n,target}} = \frac{r_{n,max} \cdot r_{n,target} \cdot t_{occupied} \cdot A}{r_{n,max} - r_{n,target}}, \tag{4}$$

where A is the total domain area.

From initial simulations of homogeneous arrays, we found that this approach resulted in a realised nucleation rate of about 80% of $r_{n,target}$ (Fig. S.1). To keep the realised nucleation rate close to the target, we increased the appearance rate by 20% to 0.018 $\mu m^{-2}s^{-1}$.

B Semiflexible microtubules

With semiflexible microtubules, we mean that the growth trajectories of individual microtubules, *in absence of interactions*, are not perfectly straight, but have an intrinsic tendency to slowly drift from their original orientation. This drift is quantified by a persistence length l_p (explained in detail in Appendix C). To implement semiflexible microtubules, we adapted an approach from Mirabet et al. [8]. We gave microtubules deflections in their growth direction at discrete points, separated by variable distances l drawn from an exponential distribution with mean $\bar{l}$. Deflection angles were drawn from a uniform interval $[-m, m]$. Angles of which the absolute value was smaller than the minimum deflection angle $q = 0.1°$ were set to zero to avoid numerical problems. As the persistence length l_p of microtubules is a function of the maximum and minimum deflection angles (see Eq. (10)), respectively m and q, and the mean deflection step size $\bar{l}$, the value for m was computed to obtain a desired persistence length given $\bar{l}$ (see next section). For $\bar{l}$ we chose a value of 1% of the desired l_p by default. This value prevents large numbers of very small deflections from resulting in many very similar trajectories that would needlessly slow down simulations and may give technical difficulties, while still keeping the step size small relative to the persistence length.

For individual microtubules averaged over sufficient length, the length of the average deflection step size $\bar{l}$ does not matter for the persistence length l_p of the total microtubule as long as the appropriate deflection angle is used. However, microtubules in a populated array interact with each other, and so there may well be a difference between many small deflections and fewer larger deflections. Therefore, we tested the effect of different step sizes for the same persistence length, for a range of step sizes feasible in the current simulation setup (Fig. S.2). For the same persistence length, the deflection step size seemed to have little effect on the alignment and orientation of arrays without bands (Fig. S.2D,E). For lower persistence lengths in particular, there seems to be a small effect on the overall density, likely resulting from differences in the rates of encounters that could lead to crossover-severing [3] or induced catastrophes (Fig. S.2A–C). Therefore, the precise way in which microtubules are flexible, may also have some impact on the array as a whole, but the magnitude of this impact on array alignment, orientation, and density is limited.

C Persistence length calculations

Persistence length l_p measures how fast the correlation between the orientation at two different points along a microtubule decays with the microtubule length between these points. Note that with l_p, we refer to the persistence length of isolated, i.e., non-interacting, microtubules. This l_p value may be affected by the crowded molecular environment of the cell cortex, but does NOT include the effect of (bundling) interactions between microtubules. We use the following definition of persistence length l_p:

$$\langle r_n \cdot r_{n+k} \rangle = e^{-\frac{L}{2l_p}}, \tag{5}$$

where $\langle r_n \cdot r_{n+k} \rangle$ is the average inner product of unit vectors r_i in the direction of the microtubule at points n and $n+k$ and L is the length along the microtubule between these two points.

Since Eq. 5 holds for any two points n and $n+k$, and we are using independent deflections, it is sufficient to look at a single deflection after length l between points n and $n+1$:

$$\langle r_n \cdot r_{n+1} \rangle = e^{-\frac{l}{2l_p}}. \tag{6}$$

Without loss of generality, we assume the arbitrary initial angle (of r_n) to be 0. For a given deflection angle ϑ (Fig. S.13A) r_{n+1} then is given by:

$$r_{n+1} = \begin{bmatrix} \cos(\vartheta) \\ \sin(\vartheta) \end{bmatrix}. \tag{7}$$

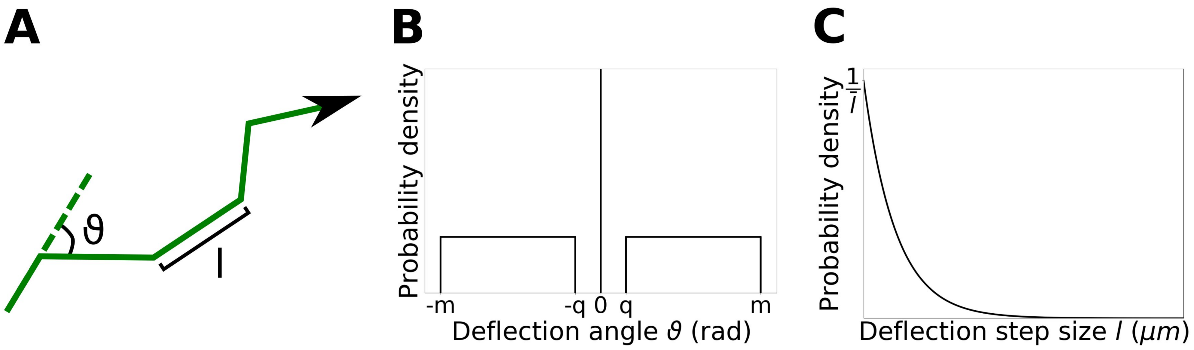

Figure S.13: Semiflexible microtubule implementation details. (A) Simulated microtubules get deflections of angle ϑ every deflection step size l. Angles in cartoon have been exaggerated for visibility. (B) Deflection angles are drawn from a uniform interval $[-m, m]$, with all angles in $[-q, q]$ set to zero. Value of q in the graph is exaggerated for visibility. (C) Deflection step sizes are drawn from an exponential distribution with average $\bar{l}$.

For the inner product in Eq. 6, we then get:

$$\langle r_n \cdot r_{n+1} \rangle = \left\langle \begin{bmatrix} 1 \\ 0 \end{bmatrix} \cdot \begin{bmatrix} \cos(\vartheta) \\ \sin(\vartheta) \end{bmatrix} \right\rangle = \langle \cos(\vartheta) \rangle. \tag{8}$$

We take deflection angles ϑ, drawn from a uniform interval $[-m, m]$, with the smallest angles ($|\vartheta| < q$) set to zero for numerical reasons (Fig. S.13B). In that case, we get:

$$\langle \cos(\vartheta) \rangle = \frac{q}{m} \cos(0) + \frac{m-q}{m} \frac{1}{m-q} \int_q^m \cos(\vartheta) d\vartheta = \frac{\sin(m) - \sin(q) + q}{m}. \tag{9}$$

Taking deflection step lengths l drawn from an exponential distribution with mean $\bar{l}$ (Fig. S.13C), we can now determine the persistence length l_p for a given value of m:

$$l_p = \langle l_p \rangle = \left\langle -\frac{l}{2\ln(\langle r_n \cdot r_{n+1} \rangle)} \right\rangle = \left\langle -\frac{l}{2\ln\left(\frac{\sin(m)-\sin(q)+q}{m}\right)} \right\rangle = -\frac{\bar{l}}{2\ln\left(\frac{\sin(m)-\sin(q)+q}{m}\right)}. \quad (10)$$

Therefore, if we draw our deflection step sizes from a distribution with average $\bar{l}$, we can solve the boundary value m of the interval from which we draw the deflection angles from the above equation for the desired persistence length l_p. This means that we can control the microtubule persistence length in our simulations using $\bar{l}$, m, and q as input parameters, with one of m and $\bar{l}$ calculated to obtain the desired l_p.

D Summary statistics

Array alignment was quantified using the planar nematic order parameter S_2 as commonly used in polymer physics and often used for quantifying cortical microtubule alignment [2, 13, 14]:

$$S_2 = \sqrt{\langle\langle \cos(2\theta) \rangle\rangle^2 + \langle\langle \sin(2\theta) \rangle\rangle^2}, \quad (11)$$

where θ is the angle of individual microtubule segments with the x-axis of the simulation domain, and double angular brackets indicate a length-weighted average over all microtubule segments. Alignment parameter S_2 is zero for a completely isotropic array and one for a perfectly aligned array (ignoring microtubule polarity).

Overall array orientation Θ was also computed as commonly used in simulation studies [2, 13]:

$$\Theta = \arctan\left(\frac{\langle\langle \sin(2\theta) \rangle\rangle}{\langle\langle \cos(2\theta) \rangle\rangle + S_2}\right). \quad (12)$$

Note that both these quantities are computed on the unrolled cylinder mantle of the simulation domain, i.e., as if it were a flat, strictly two-dimensional sheet.

Band counts were calculated as the number of bands with a microtubule density at least three times higher than the average density in the gaps. This measure was selected after testing multiple options for best representing visual inspections of array snapshots and histograms.

Averages and standard deviations of orientations from multiple simulation runs were calculated using the average and standard deviation for angular quantities. The average orientation $\bar{\phi}$ over N simulations is:

$$\bar{\phi} = \frac{1}{2}\arg\left(\frac{1}{N}\sum_{n=1}^{N} e^{i2\phi_n}\right) \quad (13)$$

where $\arg$ is the argument of a complex number, and ϕ_n is the orientation of the nth simulation. The factor 2 in the exponent and factor 1/2 before the argument correct the fact that we consider orientations without direction (so in $[0,\pi)$ rather than $[0,2\pi)$). The circular standard deviation sd_ϕ is:

$$sd_\phi = \sqrt{-2\ln\left(\left|\frac{1}{N}\sum_{n=1}^{N} e^{i2\phi_n}\right|\right)}, \quad (14)$$

where $\ln$ is the natural logarithm and the vertical bars indicate the absolute value of a complex number.

References

[1] Chakrabortty B, Blilou I, Scheres B, et al (2018) A computational framework for cortical microtubule dynamics in realistically shaped plant cells. PLOS Computational Biology 14(2):1–26. https://doi.org/10.1371/journal.pcbi.1005959

[2] Deinum EE, Tindemans SH, Mulder BM (2011) Taking directions: the role of microtubule-bound nucleation in the self-organization of the plant cortical array. Physical Biology 8(5):056002. https://doi.org/10.1088/1478-3975/8/5/056002

[3] Deinum EE, Tindemans SH, Lindeboom JJ, et al (2017) How selective severing by katanin promotes order in the plant cortical microtubule array. Proceedings of the National Academy of Sciences 114(27):6942–6947. https://doi.org/10.1073/pnas.1702650114

[4] Dixit R, Cyr R (2004) Encounters between dynamic cortical microtubules promote ordering of the cortical array through angle-dependent modifications of microtubule behavior. The Plant Cell Online 16(12):3274–3284. https://doi.org/10.1105/tpc.104.026930

[5] Jacobs B, Schneider R, Molenaar J, et al (2022) Microtubule nucleation complex behavior is critical for cortical array homogeneity *and* xylem wall patterning. Proceedings of the National Academy of Sciences 119(50):e2203900119. https://doi.org/10.1073/pnas.2203900119

[6] Lindeboom JJ, Lioutas A, Deinum EE, et al (2013) Cortical microtubule arrays are initiated from a nonrandom prepattern driven by atypical microtubule initiation. Plant Physiology 161(3):1189–1201. https://doi.org/10.1104/pp.112.204057

[7] Lindeboom JJ, Nakamura M, Saltini M, et al (2018) CLASP stabilization of plus ends created by severing promotes microtubule creation and reorientation. Journal of Cell Biology 218(1):190–205. https://doi.org/10.1083/jcb.201805047

[8] Mirabet V, Krupinski P, Hamant O, et al (2018) The self-organization of plant microtubules inside the cell volume yields their cortical localization, stable alignment, and sensitivity to external cues. PLOS Computational Biology 14(2):1–23. https://doi.org/10.1371/journal.pcbi.1006011

[9] Nakamura M, Ehrhardt DW, Hashimoto T (2010) Microtubule and katanin-dependent dynamics of microtubule nucleation complexes in the acentrosomal Arabidopsis cortical array. Nature Cell Biology 12(11):1064–1070. https://doi.org/10.1038/ncb2110

[10] Nakamura M, Lindeboom JJ, Saltini M, et al (2018) Spr2 protects minus ends to promote severing and reorientation of plant cortical microtubule arrays. J Cell Biol 217(3):915–927

[11] Schneider R, Klooster Kv, Picard KL, et al (2021) Long-term single-cell imaging and simulations of microtubules reveal principles behind wall patterning during proto-xylem development. Nature Communications 12(1):669. URL https://doi.org/10.1038/s41467-021-20894-1

[12] Tindemans S, Deinum E (2017) Corticalsim-cortical microtubule simulator. https://doi.org/10.5281/zenodo.801851

[13] Tindemans S, Deinum E, Lindeboom J, et al (2014) Efficient event-driven simulations shed new light on microtubule organization in the plant cortical array. Frontiers in Physics 2:19. https://doi.org/10.3389/fphy.2014.00019

[14] Tindemans SH, Hawkins RJ, Mulder BM (2010) Survival of the aligned: Ordering of the plant cortical microtubule array. Physical Review Letters 104:058103. https://doi.org/10.1103/PhysRevLett.104.058103

[15] Zhang Q, Fishel E, Bertroche T, et al (2013) Microtubule severing at crossover sites by katanin generates ordered cortical microtubule arrays in Arabidopsis. Current Biology 23(21):2191 – 2195. https://doi.org/10.1016/j.cub.2013.09.018
